# Intravenous Iron Versus Oral Iron Administration for the Treatment of Iron Deficiency Anemia: A Patient-Preference Study

**DOI:** 10.7759/cureus.65505

**Published:** 2024-07-27

**Authors:** Ranya Ghamri, Hadeel Alsulami

**Affiliations:** 1 Family Medicine, Faculty of Medicine, King Abdulaziz University, Jeddah, SAU

**Keywords:** anemia, iron deficiency, treatment, oral, iron, intravenous

## Abstract

Background: Intravenous iron supplementation has been reported to provide a superior safety profile and effectiveness in the treatment of iron deficiency anemia (IDA) compared to traditional oral iron supplements.

Aim: To assess preference for intravenous iron versus oral iron among patients with IDA at King Abdulaziz University Hospital, Jeddah, Saudi Arabia.

Methods: This observational cross-sectional study included 267 adults diagnosed with IDA or on treatment for IDA at King Abdulaziz University Hospital between February 2023 and March 2024. A specially modified questionnaire was used for the collection of data, which included demographic and treatment-related data. The values of the variables are presented as mean and standard deviation or median and interquartile range. Differences with an asymptotic two-tailed P-value of less than 0.05 were considered to be statistically significant.

Results: The majority of the included patients were women (95.5%), Saudi nationals (90.6%), and from the Western region (98.1%). About half of the included patients were receiving intravenous iron supplementation (51.7%), and the other half were receiving oral iron supplements (48.3%). However, the majority of the patients (74.9%) reported that they preferred intravenous iron treatment. With regard to factors that affected their preference, education level (P = 0.044), employment status (P = 0.009), and income level (P = 0.007) were identified as significant predictors. Among the patients who preferred oral iron therapy, the reason cited by the majority, that is, 79.1%, was that tablets were easier to adhere to than needles, while 50.7% stated that tablets had fewer side effects than needles and 64.2% reported a fear of needles. Among the patients who preferred intravenous iron therapy, the majority, that is, 82.4%, stated that intravenous administration was easier for them. Further, 73.5% were of the opinion that intravenous iron therapy had fewer side effects (73.5%), 27.7% reported that they were unable to swallow iron tablets, and 52.5% reported that they had difficulty remembering to take iron tablets. About a third of patients discontinued oral iron therapy due to changes in bowel habits (35%). Although 18.7% of the patients reported feeling pain with intravenous iron therapy, the majority were satisfied (79.4%) and recommended intravenous iron treatment for anemia to friends and family members (84.6%). In contrast, more than half of the patients on oral therapy were uncomfortable (56.2%) with the treatment. Further, 37.1% were not satisfied with their iron tablets, and 25.1% of patients stated that they would not recommend iron tablets for anemia treatment to their friends or family members.

Conclusion: The majority of the patients preferred intravenous iron therapy to correct IDA because oral therapy was associated with difficulties related to swallowing iron tablets and remembering to take the tablets. Although the results indicate that both therapies have similar effectiveness, patients receiving intravenous treatment appeared to be more satisfied with the treatment and recommended it to friends and family.

## Introduction

Iron is an essential component of human metabolism and has a crucial function in the production of red blood cells. Additionally, it plays a role in several other cellular activities, including DNA synthesis and cell growth [1], and is essential for the maintenance of healthy cells, skin, hair, and nails. The daily iron needed for the synthesis of red blood cells and cellular metabolism is 25 mg/day. This requirement is fulfilled by the absorption of iron from food (1-2 mg/day), the retrieval of iron from the breakdown of red blood cells by macrophages (20-25 mg/day), and the use of iron reserves (3-5 g in adults) [2]. Several factors, both non-modifiable and modifiable, have an impact on an individual’s iron balance, and they include sociodemographic characteristics such as age, sex, marital status, level of education, income, and ethnicity. Additionally, the quantity and quality of food and beverages consumed, as well as mental and physical health, medication usage, underlying medical conditions, and genetic makeup, affect iron balance [3].

Iron deficiency (ID) is a condition characterized by an inadequate amount of iron to support the normal physiological processes of tissues [4]. This condition arises because of a discrepancy between the need for iron and the amount that is consumed and assimilated. ID is linked with impaired capacity for physical labor and cognitive function, abnormal reproductive physiology, and poor pregnancy outcomes [4]. ID is commonly associated with anemia, referred to as iron deficiency anemia (IDA), which is a medical illness characterized by a deficiency in the number of red blood cells that results in an inadequate ability to transport oxygen and fulfill the body’s physiological requirements. The World Health Organization has stated that almost two billion people globally are affected by anemia, with 50% of all cases diagnosed as IDA [5]. IDA is the most widespread and typical form of micronutrient insufficiency in poor nations and is caused by prolonged and unfavorable iron imbalance [6]. Anemia caused by insufficient iron levels often results in a range of symptoms, including chronic fatigue, impaired concentration, diminished physical capacity, and a general decline in health [7]. If left untreated, IDA may progress to microcytic anemia and perhaps thrombocytosis. Typically, IDA progresses slowly and does not show any signs until the anemia gets severe [8]. The exact prevalence of IDA in Saudi Arabia has not been adequately covered by epidemiological surveys. However, there are several reports from individual institutions that focus on certain age or gender groups, and these data indicate that the prevalence of IDA in Saudi Arabia ranges from 10% to 60% [9-12].

Traditionally, the oral method of delivering iron has received significant focus [13]. However, the efficacy of readily available oral iron treatments is restricted, and their use is hindered by inadequate absorption, low adherence, adverse reactions, and significant negative consequences, such as dyspepsia and constipation [14], which diminish their efficiency. This may be partly explained by the peculiarities of the human iron regulating system, which tends to avoid high levels of iron in the long term and hinders the speedy resolution of iron shortage with oral therapy [15]. Another mode of administration is intravenous iron delivery, which is a way to receive iron through a small catheter in your vein. Relying too much on oral iron might impede the prompt delivery of intravenous iron, which has been found to be a more effective treatment in the long term. Blood transfusion for the treatment of IDA is contingent upon the degree of severity of the anemia. There are no standard criteria for blood transfusion, and practitioners use an individualized approach to determine whether their patients require transfusion, leading to the potential for unnecessary transfusions [16]. Intravenous iron treatment has been found to be a safer and more cost-effective method for replenishing hemoglobin and body iron reserves than blood transfusion [17]. Accordingly, intravenous iron sucrose formulations are now being used in several countries. Ferric carboxymaltose, another intravenous iron preparation, is currently on the market and is considered to have a good safety profile, but it is prohibitively expensive and not affordable for many individuals.

The objective of our research is to compare the efficacy and safety of intravenous iron and oral iron administration for the treatment of IDA in patients at King Abdulaziz University Hospital (KAUH), Jeddah, Saudi Arabia.

## Materials and methods

Study design, setting, and time

This observational cross-sectional study was conducted on patients diagnosed with IDA or on treatment for IDA at KAUH between February 2023 and March 2024. The required sample size was calculated using the Raosoft online sample size calculator (Raosoft, Inc., Seattle, WA), and the calculation was based on a 95% confidence interval and a 5% margin of error. The calculated sample size was 276 participants, and our sample consisted of 267 individuals.

Study participants

Our inclusion criteria were (1) age more than or equal to 18 years. This ensures that all participants are adults, facilitating informed consent and making the study results applicable to the adult population; (2) Hemoglobin (Hb) level <12 g/dL in women and <13 g/dL in men. The specific Hb level thresholds for men and women are standard diagnostic criteria for identifying anemia, ensuring that only those with clinically significant anemia are included; (3) Ferritin level <30 ng/mL. A low ferritin level is a direct indicator of ID; (4) Transferrin saturation <19%. A low percentage indicates insufficient iron supply, supporting the diagnosis of IDA. Our exclusion criteria were patients who refused to complete the questionnaire.

Data collection procedures

A questionnaire developed by the researchers was used for data collection. It was composed of five sections: The first section comprised socio-demographic data, including age, sex, nationality, marital status, education levels, employment status, region, and income level. The second section included the clinical characteristics of the patients, including height, weight, Hb level, ferritin level, years since diagnosis, and current IDA treatment. The third section constituted treatment preference and related reasons. The fourth section gathered information on the side effects experienced by the patients, and the focus of the fifth section was treatment burden and satisfaction. The data were collected using face-to-face interviews with the patients. Written informed consent was obtained from each participant to ensure they voluntarily agreed to participate in the study.

Statistical analysis

We used the Statistical Package for the Social Sciences (IBM SPSS Statistics for Windows, IBM Corp., Version 24.0, Armonk, NY) to analyze the data collected from the interviews. The Kolmogorov-Smirnov test was used to test for data distribution. The values are expressed as mean and standard deviation. The mean Hb level was compared between the two groups using the Student's t-test. An asymptotic two-tailed P-value of less than 0.05 was considered to indicate statistical significance. The data were examined with the SPSS program.

Ethical clearance

This study received the approval of the Institutional Review Board/Ethics Committee of KAUH (reference no.: 98-23). The patients’ data were securely stored and kept confidential.

## Results

Table 1 shows the demographic characteristics of the included patients. The study included 267 patients with IDA, most of whom were women (95.5%), Saudi nationals (90.6%), and from the Western region (98.1%). The majority of the patients were married (63.7%). With regard to education level, 54.7% of patients had a Bachelor’s degree and 25.8% had secondary school education. With regard to employment status, only 38.2% of the patients were employed. The common income level among 46.1% of the participants was 10000-5000 SR.

**Table 1:** Demographic characteristics of the participants (N = 267)

Characteristics		Count	%
Gender	Female	255	95.5
	Male	12	4.5
Nationality	Saudi	242	90.6
	Non-Saudi	25	9.4
Marital status	Widowed	2	0.7
	Single	78	29.2
	Married	170	63.7
	Divorced	17	6.4
Education level	Bachelor’s degree	146	54.7
	Doctorate	6	2.2
	Master’s degree	14	5.2
	Primary school	14	5.2
	Secondary school	69	25.8
	Intermediate school	18	6.7
Employment status	Not employed	157	58.8
	Retired	8	3.0
	Employed	102	38.2
Region	Southern region	4	1.5
	Northern region	1	0.4
	Western region	262	98.1
Income level (monthly)	less than 5000 SR	39	14.6
	10000-5000 SR	123	46.1
	10000-20000 SR	84	31.5
	More than 20000 SR	21	7.9

The age of the included patients ranged from 18 to 67 years (mean = 37.17 ± 11.061 years). The BMI range was 14.67-49.6, with a mean of 27.7 ± 5.92. The median ferritin level was 3.4 (2-6.8) ng/mL, and the mean Hb level was 9.4 ± 1.6 g/dL. The median number of years since the diagnosis of IDA was 3 (1-7) years, as shown in Table 2.

**Table 2:** Descriptive statistics of quantitative variables Variables with non-normal distribution are presented with the median and IQR values. BMI: body mass index; Hb: hemoglobin

Statistics	Range	Mean ± SD	Median (IQR)
Age (years)	18-67	37.17 ± 11.061	-
Height (cm)	140-180.0	157.624 ± 14.93	-
Weight (kg)	39-119	119	-
BMI (kg/m^2^)	14.67-49.6	27.7 ± 5.92	-
Ferritin level (ng/mL)	0-28	-	3.4 (2-6.8)
Hb (g/dL)	8-10.8	9.4 ± 1.6	-
Years since diagnosis with iron deficiency anemia	-	-	3 (1-7)

Table 3 shows the types of treatment and the preferred mode of iron administration. As shown, about half of the included patients were receiving intravenous iron (51.7%), and the other half were taking oral iron tablets (48.3%). However, the majority of the patients (74.9%) stated that they preferred intravenous iron treatment.

**Table 3:** Treatment type and preference

		N	%
Current iron deficiency anemia (IDA) treatment	Intravenous iron	138	51.7
	Oral iron	129	48.3
What type of iron treatment do you prefer?	Oral iron	29	10.9
	Intravenous iron	200	74.9
	No preference	38	14.2

As shown in Table 4, there was a significant association (P < 0.001) between the current IDA treatment and preferred iron treatment, with intravenous iron treatment preferred by most patients who were receiving intravenous iron (88.4%) and also the majority of the patients who were receiving oral iron (74.9%).

**Table 4:** Relation between current iron deficiency anemia (IDA) treatment and preferred iron treatment

		Current IDA treatment				Total		P-value
		Intravenous iron		Oral iron				
		N	%	N	%	N	%	
What type of iron do you prefer?	Oral iron	4	2.9	25	19.4	29	10.9	<0.001
	Intravenous iron	122	88.4	78	60.5	200	74.9	
	No preference	12	8.7	26	20.2	38	14.2	
Total		138	100.0	129	100.0	267	100.0	

Table 5 shows the relationships between the preferred mode of iron treatment and the demographic characteristics of the included patients. Education level (P = 0.044), employment status (P = 0.009), and income level (P = 0.007) were identified as significant predictors of the preferred iron treatment.

**Table 5:** Relationship between preferred iron treatment and demographic characteristics * denotes statistical significance with a P-value less than 0.05.

Characteristics		What type of iron do you prefer?						P-value
		Oral iron		Intravenous iron		No preference		
		n	%	n	%	n	%	
Gender	Female	29	100.0	191	95.5	35	92.1	0.91
	Male	0	0.0	9	4.5	3	7.9	
Nationality	Saudi	25	86.2	183	91.5	34	89.5	0.96
	Non-Saudi	4	13.8	17	8.5	4	10.5	
Marital status	Single	41	29.7	37	28.7	78	29.2	0.405
	Married	90	65.2	80	62.0	170	63.7	
	Widowed/divorced	7	5.1	12	9.3	19	7.1	
Education level	Primary	11	8.0	3	2.3	14	5.2	0.044*
	Secondary	30	21.7	39	30.2	69	25.8	
	Intermediate	7	5.1	11	8.5	18	6.7	
	Bachelor’s degree	76	55.1	70	54.3	146	54.7	
	Postgraduate	14	10.1	6	4.7	20	7.5	
Employment status	Not employed	18	62.1	118	59.0	21	55.3	0.009*
	Retired	0	0.0	6	3.0	2	5.3	
	Employed	11	37.9	76	38.0	15	39.5	
Region	Western region	134	97.1	128	99.2	262	98.1	0.201
	Other regions	4	2.9	1	0.8	5	1.9	
Income level	Less than 5000 SR	3	10.3	28	14.0	8	21.1	0.007*
	10000-5000 SR	13	44.8	94	47.0	16	42.1	
	10000-20000 SR	11	37.9	61	30.5	12	31.6	
	More than 20000 SR	2	6.9	17	8.5	2	5.3	

Table 6 shows the reasons why patients who were on oral iron treatment preferred oral treatment: tablets are easier to use than needles (79.1%), tablets have fewer side effects than needles (50.7%), and I have a fear of needles (64.2%).

**Table 6:** Reasons for preferring oral iron treatment in the oral iron group

I prefer oral iron treatment because		N	%
Tablets are easier for me to use.	Yes	53	79.1
	No	7	10.4
	Not sure	7	10.4
I have fewer side effects.	No	21	31.3
	Not sure	12	17.9
	Yes	34	50.7
I have a fear of needles.	No	43	64.2
	Not sure	5	7.5
	Yes	19	28.4

Table 7 shows the reasons why the patients on intravenous iron therapy preferred the intravenous mode of administration: intravenous iron treatment is easier (82.4%), it has fewer side effects (73.5%), I am unable to swallow iron tablets (27.7%), and I have difficulty remembering to take iron tablets (52.5%).

**Table 7:** Reasons for preferring intravenous iron therapy in the intravenous iron group

I prefer intravenous iron because:		n	%
Intravenous iron treatment is easier for me.	No	15	6.3
	Not sure	27	10.4
	Yes	196	82.4
I have fewer side effects.	No	8	3.4
	Not sure	55	22.9
	Yes	175	73.5
I am unable to swallow iron tablets.	No	155	65.1
	Not sure	17	7.2
	Yes	66	27.7
I have difficulty remembering to take iron tablets.	No	90	37.8
	Not sure	20	9.7
	Yes	125	52.5

As shown in Table 8, there was a significant difference (P < 0.001) between the two treatment groups (oral iron and intravenous iron) with regard to side effects that caused discontinuation of therapy, while 35% of patients on oral iron discontinued oral tables due to change in bowel habits, the majority (90%) of patients on intravenous iron had no side effects.

**Table 8:** Relation between current iron treatment and side effects

		Current IDA treatment				P-value
		Intravenous iron		Oral iron		
		N	%	N	%	<0.001
Side effects that cause you to discontinue the therapy, by type of anemia treatment	Various changes in bowel habits	2	1.4	45	34.9	
	Rashes/urticaria/allergy	11	8.0	1	0.8	
	Nausea/vomiting/dyspepsia	0	0	24	18.6	
	None	125	90.6	59	45.7	

Table 9 shows the perception of patients toward iron intake - both intravenous and oral iron intake. The major side effects that caused patients to discontinue therapy included changes in bowel habits (17.6%), nausea/vomiting/dyspepsia (9%), and rashes/urticaria/allergy (4.5%).

**Table 9:** Perception of patients toward iron intake

Intravenous iron treatment		n	%
Overall, how painful or physically uncomfortable were your injections for anemia treatment?	Not painful	140	52.4
	Painful	50	18.7
	Neutral	77	28.8
How satisfied were you with your injections for anemia treatment?	Satisfied	212	79.4
	Unsatisfied	2	0.7
	Neutral	53	19.9
How likely are you to recommend injections for anemia treatment to a friend or family member who may need them?	Recommended	226	84.6
	Not recommended	7	2.6
	Neutral	34	12.7
Oral iron treatment			
How uncomfortable were you with your iron tablets for anemia treatment?	Uncomfortable	150	56.2
	Neutral	46	17.2
	Comfortable	71	26.6
How satisfied were you with your iron tablets for anemia treatment?	Satisfied	95	35.6
	Unsatisfied	99	37.1
	Neutral	73	27.3
How likely are you to recommend iron tablets for anemia treatment to a friend or family member who may need them?	Recommended	138	51.7
	Not recommended	67	25.1
	Neutral	62	23.2

With regard to intravenous iron treatment, 18.7% of the patients reported feeling pain with intravenous iron therapy, but the majority of them were satisfied (79.4%) and stated that they would recommend intravenous iron administration for anemia treatment to friends or family members (84.6%).

With regard to oral iron therapy, more than half of the patients were uncomfortable with the treatment (56.2%). Further, 37.1% reported that they were not satisfied with their iron tablets, and 25.1% stated that they would not recommend iron tablets for anemia treatment to their friends or family members.

Table 10 shows that there were no significant associations between the preferred iron treatment and age of the patients (P = 0.077), BMI (P = 0.97), or the years since diagnosis with IDA (P = 0.36).

**Table 10:** Comparison of age and BMI according to preferred iron therapy BMI: body mass index

Characteristics		Mean	SD	P-value
Age (years)	Oral iron	35.93	10.330	0.077
	Intravenous iron	36.65	11.139	
	No preference	40.89	10.704	
BMI (kg/m^2^)	1.00	28.56	5.782	0.97
	Oral iron	27.471	5.82	
	Intravenous iron	29.62	6.31	
Years since diagnosis with iron deficiency anemia	No preference	3.62	5.753	0.36
	Oral iron	5.23	5.296	
	Intravenous iron	5.13	7.136	

As shown in Table 11, there was no significant difference (P > 0.05) between the two treatment groups (oral iron and intravenous iron) with regard to age, height, weight, BMI, Hb level, or ferritin level.

**Table 11:** Values of quantitative variables according to treatment type BMI: body mass index; Hb: hemoglobin

		Mean	SD	P-value
Age (years)	Oral iron	36.72	11.606	0.520
	Intravenous iron	37.59	10.552	
Height (cm)	Oral iron	158.213	6.3624	0.497
	Intravenous iron	158.725	5.9324	
Weight (kg)	Oral iron	71.64	16.975	0.246
	Intravenous iron	69.33	15.443	
BMI (kg/m^2^)	Oral iron	28.48	6.260	0.118
	Intravenous iron	27.348	5.55	
Hb level (g/dL)	Oral iron	9.4310	1.50654	0.501
	Intravenous iron	9.2978	1.70733	
Ferritin level (ng/mL) median (IQR)		Oral iron	3 (2-7)	0.963
		Intravenous iron	3.5 (2-6.6)	

## Discussion

The present study addressed the research gap in terms of data on patient preference for oral or intravenous iron treatment among individuals with IDA in Saudi Arabia. The study included 267 patients diagnosed with IDA and evaluated the effectiveness and safety of intravenous iron therapy and compared it with oral iron therapy for their treatment. In our cohort, 51.7% of the patients were receiving intravenous iron treatment, while the remaining 48.3% were taking oral iron supplements. However, a significant proportion of patients (74.9%) expressed a preference for intravenous iron therapy. Among them, most patients (82.4%) expressed a preference for intravenous iron treatment due to the ease of administration. Additionally, a significant proportion of patients (73.5%) reported that intravenous iron therapy had fewer adverse effects than other forms of iron supplementation. A notable percentage of patients (27.7%) cited difficulties swallowing iron tablets as a barrier to oral supplementation. Furthermore, more than half of the patients (52.5%) reported challenges in remembering to take iron tablets regularly. Despite 18.7% of the patients experiencing discomfort after intravenous iron therapy, a significant proportion (79.4%) expressed satisfaction and mentioned that they would recommend intravenous iron treatment to their friends or family members (84.6%). Our findings are in line with previously reported research in other countries. For example, research conducted in Nigeria by Akinajo et al. (2024) showed that intravenous iron was the favored therapeutic approach for treating IDA during pregnancy [18]. The authors outlined many benefits, such as quick and effective response to therapy, suitability for patients who cannot tolerate oral iron due to intolerance or sensory aversion, and overall improved treatment compliance [18]. In addition, according to research conducted in Australia, women showed a preference for iron infusion as a therapy for iron insufficiency, as opposed to oral iron supplements [19]. A systematic review of 15 trials further showed that the use of intravenous iron was more effective than oral administration in the treatment of IDA in pregnant women [20]. Thus, the global trend indicates a preference for intravenous iron treatment that is supported by the documented benefits of intravenous treatment over oral treatment.

With regard to the reasons for preferring a certain type of treatment, the majority of the patients (79.1%) in our study who expressed a preference for oral iron treatment reported that it was more convenient than intravenous treatment. Additionally, 50.7% of patients said that tablets had fewer adverse effects than needles. Furthermore, a significant proportion of patients (64.2%) cited fear of needles as a contributing factor to their preference for oral iron therapy. In contrast, oral medication is often discontinued by patients due to adverse effects such as alterations in bowel habits (17.6%), nausea/vomiting/dyspepsia (9%), and rashes/urticaria/allergy (4.5%). Further, a majority of patients (56.2%) experienced discomfort with oral iron therapy, while 37.1% expressed dissatisfaction with their iron pills. Additionally, 25.1% of patients stated that they would not recommend iron tablets for the treatment of anemia to their friends or family members. Similarly, previous studies have reported significant rates of non-compliance with oral iron supplements due to symptoms such as stomach pain, constipation, vomiting, and weight gain [19]. In fact, approximately 60% of those who use oral iron supplements have gastrointestinal adverse effects [21], and up to 50% of patients receiving oral iron therapy for IDA do not adhere to their treatment plan due to gastrointestinal problems, resulting in the persistence of their condition [22]. In contrast, individuals who receive intravenous iron infusions had a reduced incidence of these adverse effects [21], including nausea (1.6% vs. 4.9%), vomiting (1.0% vs. 6.8%), stomach discomfort (1.3% vs. 7.9%), and diarrhea (0.9% vs. 8.3%) [23]. Since intravenous iron does not travel through the gastrointestinal tract, it is believed that any gastrointestinal adverse effects reported are mostly caused by the direct interaction of iron with the gastrointestinal environment when taken orally [23]. However, intravenous administration may cause severe and, sometimes, life-threatening adverse effects, such as hypersensitivity and anaphylaxis [24]. Thus, it should be prescribed with caution. According to our results, education level (P = 0.044), work status (P = 0.009), and income level (P = 0.007) were shown to be significant predictors of preference for iron therapy.

Some limitations of the present study need to be explained. First, the limited sample size was insufficient to determine the safety of intravenous iron delivery, particularly in relation to the rare but severe adverse effects of this mode of treatment. Further research is required to investigate other significant effects, such as the consequences of oxidative stress resulting from iron and its lasting impacts, in order to confirm the superiority of intravenous iron over oral medication in the treatment of IDA. Despite these limitations, this was the first study to compare the preferences of patients with IDA for oral and intravenous iron therapy in Saudi Arabia, so the findings are important for understanding patient preferences, treatment outcomes, and optimal therapy strategies in this region.

## Conclusions

The majority of patients preferred intravenous iron administration for the treatment of IDA. This may account for the swift rise in the use of intravenous iron. However, the qualitative data collected here indicate that there are several similarities in the effectiveness of both treatments. Based on the findings from our cohort and previous studies, we recommend that the more affordable and safer option, that is, oral iron supplementation, be prioritized as the initial treatment, with intravenous administration provided in cases of intolerance to oral, poor absorption or need to increase the iron level quickly due to anemia or pregnancy. Moreover, additional study is necessary to ascertain the effectiveness of intravenous iron treatment, especially in individuals with IDA.
